# Development of Flow-Through Cell Dissolution Method for In Situ Visualization of Dissolution Processes in Solid Dosage Forms Using X-ray μCT

**DOI:** 10.3390/pharmaceutics14112475

**Published:** 2022-11-16

**Authors:** Niloofar Moazami Goudarzi, Aseel Samaro, Chris Vervaet, Matthieu N. Boone

**Affiliations:** 1 Department of Physics and Astronomy, Radiation Physics, Ghent University, Proeftuinstraat 86/N12, 9000 Gent, Belgium; 2 Centre for X-ray Tomography (UGCT), Ghent University, Proeftuinstraat 86, 9000 Gent, Belgium; 3 Laboratory of Pharmaceutical Technology, Department of Pharmaceutics, Ghent University, Ottergemsesteenweg 460, 9000 Gent, Belgium

**Keywords:** dissolution, μCT, flow-through cell method, contrast agent, 3D printing, sustained release

## Abstract

Visualization of the dynamic behavior of pharmaceutical dosage forms during the dissolution process offers a better understanding of the drug release mechanism, enabling the design of customized dosage forms. In this study, an X-ray tomography-based approach is proposed to monitor and analyze the dynamics of the structure at the pore scale level during the dissolution process. A flow-through cell dissolution apparatus was developed, capable of mimicking the standard in vitro dissolution process, which can be easily positioned in an X-ray tomography setup. The method was utilized to study the dissolution of a Capa^®^ (polycaprolactone)-based sustained-release 3D printed tablet. The impact of the flow rate on the active pharmaceutical ingredient (API) release rate was studied and 16 mL/min was selected as a suitable flow rate. Furthermore, cesium chloride (CsCl) was used as a contrast agent to increase the contrast between the sample and the dissolution medium. Data obtained with this novel technique were in a good agreement with the released drug rate acquired by the standard in vitro dissolution test (the similarity factor ($f_{2}$) = 77%). Finally, the proposed approach allowed visualizing the internal structure of the sample, as well as real-time tracking of solution ingress into the product.

## 1. Introduction

Following tablet development, multiple quality-control tests are performed on a product before being approved for commercialization [1,2]. In vitro dissolution testing is a key quality control tool to measure the extent and the rate at which an active pharmaceutical ingredient (API) is released from the dosage form into a liquid medium under standardized conditions of volume, temperature, agitation, flow rate, and solvent composition. The release rate of API from a solid dosage form is controlled by different properties, such as the physicochemical properties of the API and excipients, and morphological properties of the final dosage form, such as porosity and density distribution. Hence, detailed information on fluid diffusion patterns into the tablet as well as the internal structure of the product (such as pore size, number of pores, and tortuosity of the matrix) is necessary to obtain a comprehensive knowledge of the drug release mechanisms from different types of formulation, and to use this knowledge to design customized dosage forms via adapting the processing technique and the formulation composition [3,4,5].

Conventional in vitro dissolution tests, such as the paddle and basket dissolution method [6], can only quantify the drug release rate and do not provide spatial information about the product during the dissolution process. Different methods have been developed to study the internal structure of tablets, such as mercury porosimetry and gas adsorption [7,8,9]. These techniques are destructive and can not be used to investigate the dynamic property of the product during the dissolution process. Thus, there is a growing demand for developing non-destructive techniques to visualize and characterize the inner structure of solid dosage forms during the dissolution process. In situ characterization of the dissolution process of dosage forms has been accomplished using UV-Visible microscopy [10] and optical microscopy [11]. However, measurements have so far been limited to the sample’s surface. Magnetic resonance imaging (MRI) has been utilized for three-dimensional in situ characterization of the dissolution process [12,13,14,15]. Although it can be carried out continuously in a standard flow-through cell apparatus (USP 4), the method suffers from low spatial resolution and relatively long echo times [16,17].

High-resolution X-ray computed tomography (μCT) is a non-invasive technique that can be utilized to visualize both the external and internal structure of the object at resolutions down to a few hundred nanometres. It allows for reproducing a complete three-dimensional (3D) representation of the object by combining the information of the regular two-dimensional radiographic projections taken from different angles. Over the last two decades, μCT has become an established technique in various research domains such as medicine [18], geoscience [19], material science [20], wood technology [21], soil science [22] as well as industrial applications [23,24,25]. Specifically in porous media, it is frequently applied to obtain quantitative information on density distribution, porosity, and properties of the pores, such as geometry, size, surface area and connectivity [26].

In the pharmaceutical industry, μCT has previously been employed to evaluate the internal structure, density variations [27,28], ingredient distribution [29], the thickness of coating layer [30,31], and the porosity of the tablets and granules [32,33]. Several studies investigated the application of μCT to monitor the dynamic changes inside the tablets during the dissolution process [3,34,35,36,37,38,39]. In these works, the dissolution test was performed using the paddle/basket dissolution apparatus, where tablets were extracted and dried at predefined time steps and then analyzed by μCT to assess the structural change of the samples. However, it is very likely that the morphology of the internal structure changes during the dehydration process. Additionally, as different tablets were used for different time steps, inter-sample variability undoubtedly affects the correlation between release data and physical characteristics of the matrix structure as a function of time. In another work, Gioumouxouzis et al. [40] employed 4D-CT to qualitatively study the morphological changes of 3D printed dosage forms composed of three compartments: an upper and lower layer made of inert polymer polylactic acid (PLA), and an intermediate layer consisting of hydrochlorothiazide (HCTZ), polyvinyl alcohol (PVA) and mannitol blend. The in vitro dissolution test was performed with the paddle dissolution method. At each time step, they extracted the sample from the reservoir and then placed it on the scanner’s stage for further imaging. After each scan, they returned the sample to the reservoir. They reported a partial detachment of the outer PLA component and attributed this to the frequent removal of the specimen from the solution and its resulting mechanical stress. Young et al. [41] analyzed the internal structure of ferrous sulphate-Ferrogradumet^®^ inert matrix tablets upon dissolution by μCT. To simulate the Pharmacopeia dissolution test, they used a reservoir of 900 mL capacity upon a magnetic stirrer. The samples were glued on top of a Styrofoam, and the foam was placed inside an 80 mL polymer tube. The tube was then positioned inside the reservoir to conduct the dissolution experiment. Every time imaging was due, the tube was pulled out of the medium. After removing excess fluid and covering the tube with a parafilm layer, it was placed on the scanner stage for further imaging. The images revealed slower diffusion of the fluid through the lower face of the tablet, which was suggested to be due to the gluing of the sample.

In this work, we introduce a dissolution method that enables us to evaluate the internal structure of the pharmaceutical dosage forms during the dissolution process using μCT. An in vitro dissolution experiment with X-ray tomography requires customized apparatus capable of imitating the in vitro dissolution process while obeying the constraints of the imaging technique. To be used at the μCT setup, this device is bounded to several physical limitations, such as size, shape and material selection. Furthermore, to minimize motion artifacts, the sample needs to be positioned stably. Given all these constraints, a flow-through cell method was designed and developed that simulates the in vitro dissolution process obtained with well-established methods, and can be used at a μCT system. To assess the applicability of the method, a model 3D printed tablet comprising a mixture of insoluble polymer and soluble drug particles was utilized. Due to the low difference in X-ray attenuation coefficient between the medium and the specimen, two different contrast agents were evaluated to increase the attenuation coefficient of the dissolution medium. The impact of the contrast agents and the flow rate on the API release ratio from the tablet was studied. μCT images were captured, and penetration of the medium to the sample was visualized.

Moreover, a Capa^®^-based sustained release 3D printed tablet was used in this study, as this is a non-swellable and non-eroding dosage form, which is the easiest formulation to monitor during the dissolution process. Furthermore, 3D printing has recently gained massive attention in the pharmaceutical field to prepare personalized medicine in which the dose can be easily adapted based on the patient’s need by adjusting the printing setting, the shape and the size of the dosage form [5,42,43,44,45]. This study used the fused filament fabrication (FFF) 3D printing technique. During FFF, filaments prepared by hot-melt extrusion were used as feedstock material. The filament passes through the pinch rolls of the printer towards the extrusion nozzle, which is heated to the desired temperature to melt the filament and deposit it by a computer-guided movement on the building platform layer by layer.

The remainder of this paper is organized as follows: In Section 2, we describe the experimental preparation and apparatus, as well as imaging and analyzing methods. We represent the results together with our interpretation of the results in Section 3. Finally, we conclude this work in Section 4.

## 2. Methods and Materials

### 2.1. Materials

Metoprolol tartrate (MPT) (Utag, Netherlands) was used as a model drug in combination with the hydrophobic polymer Capa^®^ 6506. Capa^®^ 6506 was kindly donated by Perstorp (Warrington, UK). Capa^®^ 6506 is a polycaprolactone grade with a molecular weight of 50,000 Da, a melting temperature of 58–60 ${}^{{}^{\circ}}$C and a glass transition temperature of −60 ${}^{{}^{\circ}}$C.

Cesium chloride and potassium iodide (KI) with 99.9% and 99.5% purity, respectively, were purchased from Carl Roth (Karlsruhe, Germany). These were further used as the contrast agent for the X-ray tomography.

### 2.2. Tablet Preparation

Physical mixtures of 40% *w*/*w* drug load were extruded using a Prism Eurolab 16 co-rotating twin-screw extruder (Thermo Fisher Scientific, Germany) with a barrel length of 25 L/D (where L is the axial screw length of the machine and D is the inner bore diameter corresponding to one of the screws) and equipped with a custom-made die with a diameter of 1.75 mm. The feeding rate, screw speed and barrel temperature were set at 0.30 kg/h, 100 rpm and 100 ${}^{{}^{\circ}}$C, respectively. A rotating device with controlled speed was positioned at the end of the extruder and coupled with a spool to collect the extruded filament at room temperature.

Filaments that were prepared by hot melt extrusion were further used to print tablets using a Prusa i3 MK3S printer (Prusa Research, Prague, Czech Republic). A printing temperature of 110 ${}^{{}^{\circ}}$C was used, in combination with a layer height of 0.25 mm, 2 external shells, 100% rectilinear infill, 10 mm/s printing speed for all layers and a nozzle diameter of 0.4 mm. No raft was used, and the building platform temperature was set to 20 ${}^{{}^{\circ}}$C. The printed tablets had diameter equal to 10 mm, height of 2.8 mm, and weight of 164 ± 1.69 mg. Moreover, Differential Scanning Calorimetry (DSC) after extrusion and after tableting confirmed that API was stable and 100% crystalline [5]. In addition, a drug assay of the 3D printed tablets confirmed that the drug content was between (98.5–101% *w*/*w*).

### 2.3. Flow-Through Cell Dissolution Apparatus

**Figure 1:**
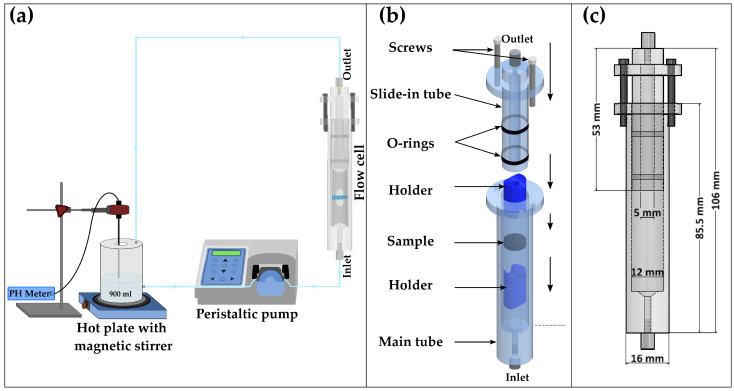
(**a**) Schematic representation of the flow-through cell dissolution apparatus; (**b**) different components of the flow cell; (**c**) 2D CAD model of the flow cell including the dimensions.

A custom-built flow-through dissolution system was developed consisting of a flow cell, liquid reservoir, peristaltic pump (Vantage 3000 P EZ- Verder Ltd.-Castleford-UK), pH meter (PH3310- WTW- Weilheim in Oberbayern-Germany) and a hot plate with a magnetic stirrer (Figure 1a).

The flow cell was constructed in polymethyl methacrylate (PMMA) due to its relatively low X-ray attenuation, which ensures that sufficient X-ray flux reaches the detector. As it is illustrated in Figure 1b, the flow cell is composed of a main cylindrical body, a slide-in tube and two holders. The dimensions of the assembled flow cell are indicated in Figure 1c. The height of the main body is 85.5 mm with 12 mm inner diameter and 16 mm outer diameter. The height, outer and inner diameters of the slide-in tube are 53 mm, 11.5 mm and 5 mm, respectively. To prevent leakage from the system, two grooves were made on the slide-in tube, and then two rubber rings were placed inside the grooves, forming a seal at the interface of the main body and the slide-in tube. The inlet and outlet of the flow cell were equipped with a threaded male M3 push-in fitting with 4 mm outer diameter. To position the tablet inside the flow cell, two cylindrical holders with a conical-shaped cut-out are placed on the bottom and top of the sample.

The flow-through cell apparatus worked in a closed-loop configuration, in which the medium was pumped in a circle and not replaced by a fresh medium. The formation of air bubbles was observed during the preliminary tests. Former studies indicated that, without any measures to prevent this, air bubbles can change the water absorption and disintegration rate of tablets by adhering to their surface, thus altering their contact surface with the media [46,47]. In this study, to avoid this, vacuum degassing was performed to deaerate the medium. A vacuum chamber with a cylindrical shape and capacity of approximately 3 L was designed and fabricated in PMMA. The chamber was equipped with a valve on top, allowing the connection to the vacuum pump. It also worked as the reservoir for the flow-through cell system, giving the advantage of avoiding bubble formation in the media when transferring the liquid from one container to another. The vacuum chamber also consisted of one inlet at the top and two outlets on the side, at the height of 5 cm from the bottom. One outlet was designed for making a connection with the flow cell, and the other for collecting samples from the reservoir for further analysis. The inlet was made at the top of the reservoir to remove the introduced air bubbles from the system. When a droplet falls on the gas–liquid boundary, air bubbles are separated from the droplets due to the buoyancy effect [48]. Moreover, the pH of the system was monitored during the dissolution process.

### 2.4. In Vitro Dissolution Experiments

#### 2.4.1. Paddle Dissolution Method

A USP paddle dissolution apparatus (VK 7010, VanKel Industries, NJ, USA) was used to study the release rate of MPT from the 3D printed dosage forms. The experiments were performed at 37 ± 0.5 ${}^{{}^{\circ}}$C, and the paddle rotated at 100 rpm. Dissolution vessels were filled with 900 mL of the medium. The tablet was placed inside a cylindrical basket to avoid floating, and the basket was inserted into the dissolution vessel, immediately after running the dissolution apparatus. A sample of 5 mL was taken from the dissolution medium at predetermined time points (i.c., 0.25, 0.5, 0.75, 1, 1.5, 2, 2.5, 3, 3.5, 4, 5, 6, 7, 8 and 10 h). The amount of dissolved active ingredient in the samples was measured using the UV/Vis spectrometer. All experiments were conducted in triplicate.

#### 2.4.2. Flow-Through Cell Dissolution Method

The reservoir was filled with 900 mL of the dissolution medium, and it was connected to the vacuum pump to remove the dissolved gas. The deaeration took about one hour at a pressure close to 10 mbar. Next, the vacuum chamber was disconnected from the vacuum pump and was returned to atmospheric pressure. Then, the temperature of the medium was raised and maintained at 37 ± 0.5 ${}^{{}^{\circ}}$C using the hot plate. To assemble the flow cell, the sample was positioned between the two sample holders inside the main body. The slide-in tube was then inserted into the main body, and it was fixed via two screws, to avoid any movement as a result of pulsatile flow through the system. Next, the reservoir was connected to the flow cell, and the peristaltic pump was positioned between these two. When the temperature reached the test condition, the pump applied a controllable flow to the flow cell, from bottom to top. During the dissolution process, the liquid inside the reservoir was stirred using the magnetic stirrer, and a sample of 5 mL was withdrawn at predefined time steps (similar to Section 2.4.1). These samples were analyzed by UV/Vis spectrometer to measure the released API. All experiments were conducted in triplicate.

#### 2.4.3. UV Spectrophotometry

UV absorbance measurements of all samples were performed using a UV-1650PC spectrometer (Shimadzu Benelux, Antwerp, Belgium). First, the wavelength with maximum intensity ($\lambda_{max}$) was determined and was further used to record the absorbance values of the samples. Then, different solutions of known MPT concentrations, ranging from 5 to 40 μg/mL, were prepared. Their absorbance (at $\lambda_{max}$) was measured and a linear calibration curve of the absorbance versus concentration was created. Using this calibration curve, the concentration of dissolved active ingredient in the dissolution experiments was determined by converting the absorbance into concentration.

**Figure 2:**
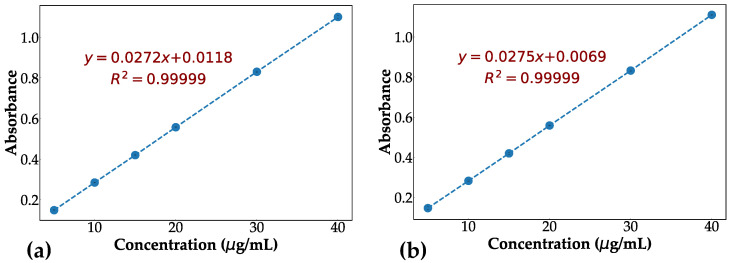
The calibration curve for determination of MPT in (**a**) phosphate buffer and (**b**) CsCl brine. The equation of the fitted line and the coefficient of determination ($R^{2}$) are demonstrated in each plot. The wavelength at which the drug content was measured in phosphate buffer, and CsCl brine was 222 nm and 223 nm, respectively.

The UV spectra of MPT in phosphate buffer and CsCl brine showed that the drug absorbed appreciably at 222 nm and 223 nm, respectively. Therefore, these wavelengths were selected as the detection wavelength ($\lambda_{max}$) to determine the API concentration in the dissolution medium. The calibration curve was constructed for each dissolution medium, as demonstrated in Figure 2.

### 2.5. High-Resolution X-Ray Tomography (μCT)

μCT is a non-destructive imaging technique to investigate internal features and dynamic processes in materials. The X-ray beam passing through a material will be attenuated due to the interaction of the photon with the matter. The amount of X-rays absorbed by an object is defined by Beer’s law as a function of the attenuation coefficient and thickness of a material. For a monochromatic X-ray beam passing through a single material, it is determined using the following equation:

(1) $$I_{out}=I_{in}e^{-\mu T},$$

where $I_{out}$ and $I_{in}$ are the intensity of the attenuated radiation and the intensity of the incident radiation, respectively. Here, μ is the linear attenuation coefficient of the material, and T is the thickness of the material. μCT combines a set of radiographs taken from different angles to obtain a 3D image of the sample under investigation. The resulting image consists of voxels with a gray value representing the local X-ray attenuation coefficient in the object. This enables us to monitor and quantify the composition within the object.

#### 2.5.1. μCT Set-Up

The Environmental Micro-CT (EMCT) scanner of the Ghent University Centre for X-ray Tomography was used for imaging [49]. Unlike conventional μCT systems, the sample remains fixed while the X-ray source and detector rotate around the sample in a horizontal plane. This unique system is designed specifically to conduct dynamic high-resolution X-ray computed tomography (4D-μCT) experiments, in which fluid flow or pressure are involved, as the fast and continuous rotation of the sample is difficult to achieve in the presence of cables and tubing. EMCT is equipped with a directional target X-ray source with the best resolution of 5 μm that can reach a voltage up to 130 kV and a maximum power of 39 W. Its detector is a CMOS flat panel with a CsI scintillator of 1316 by 1312 pixels with a 100 μm pitch. This system is optimized for high scanning speed, with the highest speed of 12 s for a full rotation, with the best resolution limited to about 15 μm at these short acquisition times [50].

**Figure 3:**
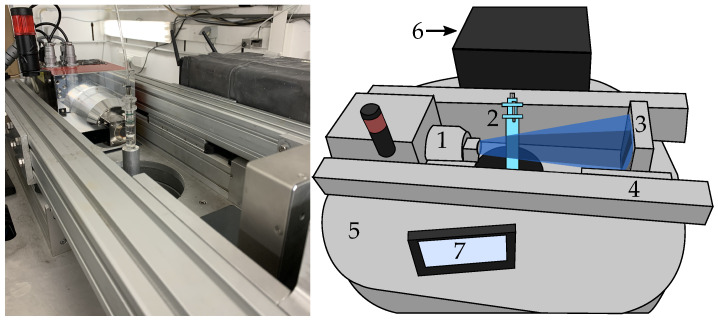
Picture and schematic representation of UGCT’s EMCT scanner: (1) the X-ray source, (2) the flow cell, (3) the detector, (4) the magnification axis, (5) the rotation stage, (6) the computer system with its touch-screen control panel (7). The flow cell is mounted on a stand which moves into the FOV of the scanner from below. The source and detector rotate around the sample, while the sample remains fixed.

To capture the X-ray images, the flow cell was positioned in the center of scanner’s field of view (FOV), as it is illustrated in Figure 3. Before dissolution started, a higher resolution pre-scan was taken to determine the static morphology and pore structure of the sample. It was acquired with a higher frame averaging and more projections for a full rotation. Then, X-ray μCT scans were acquired with higher speed and lower resolution when the flow cell is filled with the medium and at several time steps ranging up to 8 h of the dissolution process. In all scans, the tube was operated at a power of 8 W and voltage of 80 kV with an exposure time of 100 ms per image for a full 360${}^{\circ }$ rotation. The voxel size and other relevant scanning parameters are indicated in Table 1.

#### 2.5.2. Contrast Agent

The attenuation coefficient of a material (μ in Equation (1)) depends on the density and the atomic number of the material and on the energy of the incident photons. Drug dissolution from dosage forms composed of metallic API can be visualized and characterized using μCT [41]. However, for non-metallic APIs with similar atomic number and density to the dissolution medium, it is impossible to distinguish between the sample and the medium [51]. In this study, the most abundant element in the model tablet is carbon, while oxygen is the major element in the dissolution medium (in this work, phosphate buffer). Since carbon and oxygen have a nearly equal atomic number, they similarly attenuate the X-ray beam, resulting in a low contrast between the solvent and the sample. Therefore, a contrast agent consisting of an element with a high atomic number is needed to enhance the attenuation of either the fluid, the matrix material, or in some cases, the active product.

In this work, CsCl and KI were employed as the contrast agent to increase the attenuation coefficient of the dissolution medium. A total amount of 50 gr KI and 80 gr CsCl was dissolved in sufficient phosphate buffer (pH = 6.8) to make 1000 mL of 5% (*w*/*v*) KI brine (pH = 6.5) and 8% (*w*/*v*) CsCl brine (pH = 6.6 ), respectively.

#### 2.5.3. Image Analysis

Tomographic reconstruction of all scans was performed using Octopus Reconstruction, an in-house developed software package [52]. Further image processing was conducted utilizing Octopus Analysis [26], ImageJ/Fiji, and a custom-developed Python script as explained in the following paragraphs. Additionally, volume visualization and 3D rendering was carried out using VGSTudio MAX 3.2 (Volume Graphics GmbH, Berlin/Heidelberg, Germany).

First, the total porosity of the sample was determined as the ratio of the tablet’s pore volume to its total volume. In this regard, the reconstructed volume of the tablet at the dry state was loaded in Octopus Analysis. After applying a bilateral filter and segmenting using a manually selected threshold, the surrounding area was removed. Then, the pore space was segmented by adjusting the threshold, and the porosity was calculated.

Next, the penetration of the medium into the polymer matrix was visualized. In this respect, a mask was made to select the tablet’s matrix ( the dosage form, excluding the pore space) as the volume of interest (VOI). As the structural change of the tablet was negligible, the dry scan was used to create the mask for the images obtained during the dissolution. The data of the dry scan were registered and resampled to the fast scans’ data set. Next, it was treated with a bilateral filter and segmented by manual thresholding, resulting in a mask to identify the tablet’s matrix. The mask was applied to the data sets obtained at the different time steps of the dissolution process using a Python script. To visualize the progress of water into the matrix, the histogram of the tablet’s matrix, which is an approximate representation of the distribution of voxels’ value, was studied. A single threshold was adjusted to segment the wetted matrix by evaluating the variation in the histogram over time.

Finally, the rate at which water penetrated into the tablet was measured during the dissolution process. The penetration rate was defined as the number of wetted voxels divided by the total number of voxels of the VOI. In this work, the K-Nearest Neighbor (KNN) algorithm was performed by the Scikit-Learn library to discriminate the wetted voxels from the dry voxels [53]. KNN is a classification algorithm that classifies an input X based on its nearest K neighbors in the training data [54] (p. 39). To classify a new voxel, the algorithm finds K nearest voxels in the training data set based on a predefined distance (i.e., Euclidean distance) and other parameters. Then, the voxel is classified based on the majority class presented in that neighborhood. In this study, the images acquired at the first time step and the last time step were considered as training samples. Coordinate, gray value, and Euclidean distance to the closest background voxel for each of the foreground voxels were used as input features. To validate the classification, 10% of the training samples were randomly chosen. Based on the performance and required training time, the number of neighbors was determined to be 10, resulting in the test error of about 1.88%. The training was performed assuming all voxels in the first step and in the last step were completely dry and hydrated, respectively. Finally, the model was applied to classify each voxel in the 3D image for each time step.

## 3. Results and Discussion

### 3.1. Fine Tune Flow-Through Cell System

**Figure 4:**
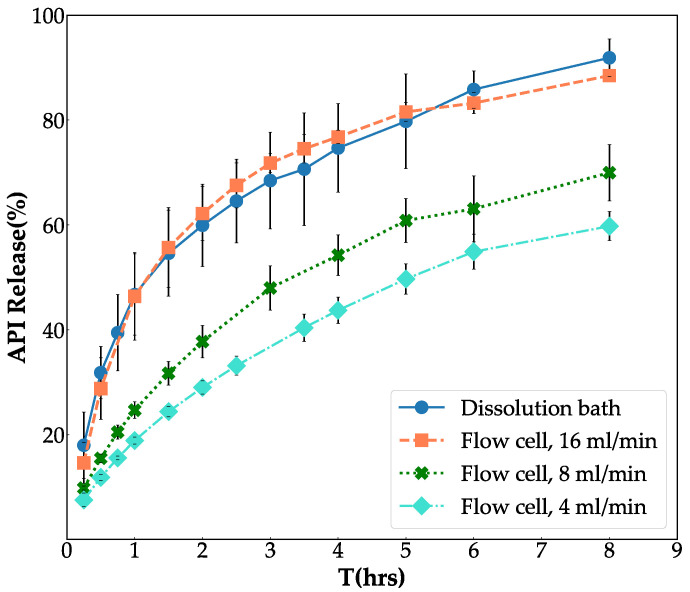
Release profile obtained by the paddle dissolution method in comparison with that obtained by the flow-through cell method with different flow rates (16 mL/min, 8 mL/min, and 4 mL/min). All tablets were dissolved in phosphate buffer.

The impact of the flow rate on the dissolution of API from the tablet’s matrix was studied, and the resulting release profiles were compared to that obtained by the paddle dissolution method. First, as described in Section 2.4.1, the dissolution was conducted using the paddle dissolution method in phosphate buffer. Then, the flow-through cell method was performed with flow rates equal to 4, 8, and 16 mL/min in phosphate buffer (Section 2.4.2). Figure 4 illustrates obtained release profiles with both systems. It indicates that an increase in the flow rate enhanced the dissolution rate. Moreover, slight difference can be seen between the released profile of the API in the flow cell with 16 mL/min flow rate and that obtained in the dissolution bath. The dissolution profiles of the API released from tablets in the flow cell with a specific flow rate were compared with that obtained by the paddle dissolution method by calculation of the similarity factor ($f_{2}$) [55], and the results are listed in Table 2. This analysis suggested that there is an excellent agreement between the dissolution profile obtained by the flow cell with the flow rate equal to 16 mL/min and that obtained by the paddle dissolution method ($f_{2}$ = 77%).

### 3.2. In Vitro Validation of Contrast Agent

**Figure 5:**
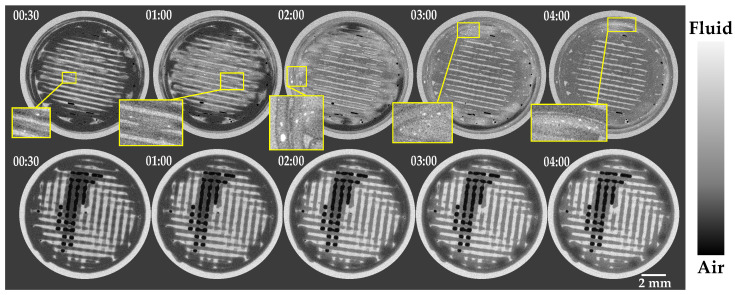
2D cross-sectional images of a tablet in KI brine and in CsCl brine are displayed in the top and bottom row, respectively. The time points at which the scans were taken are mentioned for each slice. Yellow rectangles highlight the location of crystallized KI within the sample.

In order to examine the adequacy of the contrast agents, dissolution experiment was performed using the flow-through cell method, one in CsCl brine and the other in KI brine, as described in Section 2.4.2. X-ray images were acquired at different time steps during the dissolution (as described in Section 2.5.1). Qualitative evaluation of the reconstructed 3D images supports the application of both CsCl and KI as a contrast agent to track the water ingress into the sample (Figure 5). On the other hand, the results obtained from the sample in KI brine suggested the tendency of KI to start nucleating inside the tablet. As can be seen in the top row of Figure 5, bright spots emerged within the wetted matrix of the tablet during the dissolution and faded after a while. Consequently, CsCl was selected for further evaluation, as using KI has a negative impact on the image of the tablet due to the crystallization of the salt. First, experiments also suggested that UV absorption measurements were highly affected when using KI as the contrast agent.

**Figure 6:**
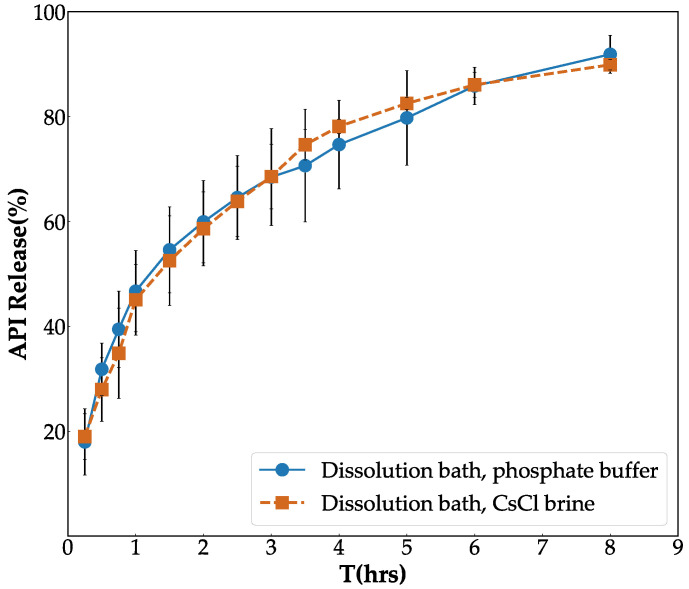
Release profile obtained by the paddle dissolution method in different solvents: phosphate buffer and CsCl brine.

To investigate the precise impact of CsCl on the dissolution profile of API from the tablets, two paddle dissolution tests were performed, one using CsCl brine as the dissolution medium and the other in phosphate buffer. All studies were conducted in triplicate, as described in Section 2.4.1. The UV spectrophotometer was used to measure the percentage of the released API at a wavelength ($\lambda_{max}$) of 222 nm and 223 nm for CsCl brine and the phosphate buffer, respectively. The dissolution profiles of the tablets in different media are presented in Figure 6. The $f_{2}$ value was also calculated to compare the release profiles of tablet in different media, resulting in a value about 78%. This analysis indicated that the release profile in CsCl brine was not significantly different; thus, it is well suited as a contrast agent.

**Figure 7:**
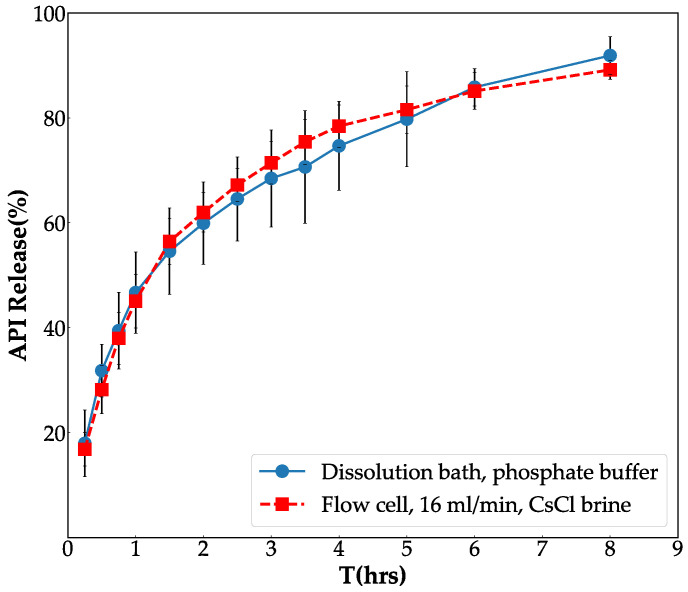
API release profiles obtained by the paddle dissolution method in phosphate buffer, and that obtained by the proposed dissolution method with a flow rate of 16 mL/min in CsCl brine.

Considering the previous experiment (Section 3.1), 16 mL/min and CsCl were chosen as the suitable flow rate and the contrast agent, respectively. The dissolution profile obtained by the flow-through cell method with 16 mL/min in CsCl brine and that obtained from the paddle dissolution method in phosphate buffer are plotted in Figure 7. The closeness between the two profiles ( with $f_{2}$ = 77%) confirms the competence of the proposed flow-through cell dissolution method to mimic the in vitro dissolution process conducted by the paddle dissolution method.

### 3.3. μCT Scanning

A flow-through cell dissolution experiment was performed with the flow rate equal to 16 mL/min in CsCl brine. As described in Section 2.5.1, imaging was performed via high-resolution X-ray computed tomography. The total porosity of the tablet in the dry state was calculated as 23.24% ± 0.60% (the standard deviation was provided by scanning three different tablets).

**Figure 8:**
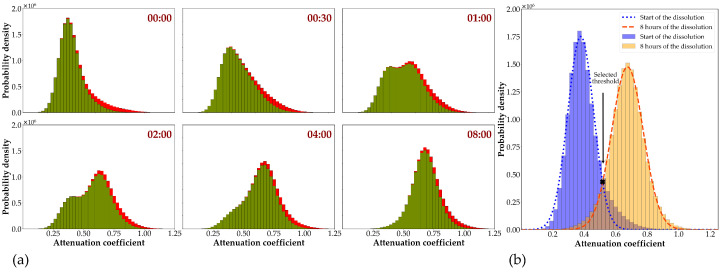
(**a**) Histogram of the X-ray attenuation coefficient at six different time steps of the dissolution process. The full histogram represents the tablet’s matrix, and the red part is what has been omitted by the shrinking. The time points at which the scans were taken are mentioned at the upper right corner of each graph; (**b**) the histogram of the attenuation coefficient for the VOI at the time that the dissolution medium filled the flow cell (blue) and for the scan taken at 8 h of the dissolution process (orange). The blue dotted line and orange dashed line represent the Gaussian distributions fitted on each histogram, respectively. The intersection of the two Gaussian distributions is used to segment the wetted matrix.

**Figure 9:**
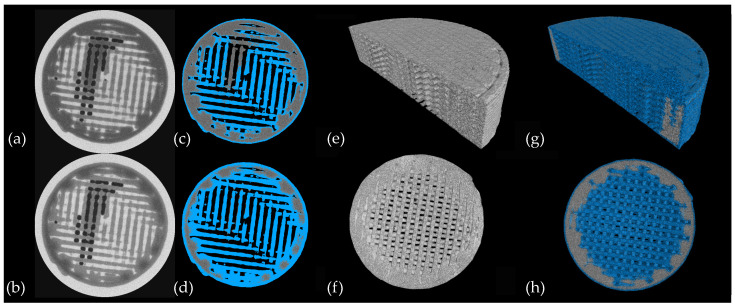
(**a**,**b**): The horizontal slice through the micro-CT volume of the tablet at 1 h and 4 h of the dissolution are represented in, respectively; (**c**,**d**) The segmented wetted matrix overlaid on the same slices (after removing the area around the VOI). 3D renderings of tablet’s matrix, axial (**e**) and radial (**f**) cross-sections before dissolution, axial (**g**) and radial (**h**) cross-section after 4 h of dissolution.

Fluid ingress into the matrix was visualized as discussed in Section 2.5.3. To this end, the tablet’s matrix was first segmented and considered as the VOI, and the histogram of VOI’s voxel value was assessed. The histograms at different time points are represented in Figure 8a. As can be seen, the shape of the histogram changed during the dissolution process due to the penetration of the fluid into the tablet. At the early time points, one peak with a low attenuation can be observed, and as time progressed, one extra peak with a higher attenuation appeared in the histogram. An increase in the area under the second peak was noted due to an increase in the content of CsCl following the penetration of the medium into the tablet’s matrix. Gradually, the quantity of the voxels related to lower attenuation decreased over time and vanished after full penetration of the brine into the sample. To segment the wetted matrix, the intersection of the first and the last histogram was determined and served as the threshold. However, the histogram of the VOI at the first time point was noticed to be skewed to a higher gray value (the full histogram in Figure 8a). This might be due to the penetration of the solution into the sample during the imaging. As the histogram of the first step must be a good representation of the intact matrix, the VOI was shrunk to remove the wetted part in the first time step. For the sake of consistency, VOIs at all time steps were shrunk, and the resulted histograms are plotted by green bars in Figure 8a. Finally, the value at which the fitted probability distribution on the histogram (green bars) of the first and the last time steps intersect (Figure 8b) was determined and employed as the threshold value to visualize water penetration. The water penetration into the tablet at times of 1 h and 4 h of dissolution is illustrated in Figure 9.

**Figure 10:**
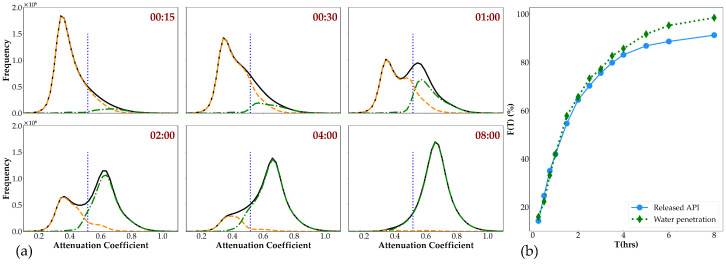
(**a**) These six graphs show six different time points of the experiment, and the time steps are mentioned in the upper right corner of each graph. The histograms for the two phases in the tablet’s matrix during the dissolution are plotted based on the KNN classification algorithm, with orange (dashed line) representing the intact matrix, green (dash-dotted line) representing the wetted matrix, and black (solid line) representing all phases combined. Each graph includes the threshold value determined in Figure 8b, represented by the blue dotted line; (**b**) API release profile measured by UV spectrophotometer represented by blue (solid line), and water penetration profile determined from X-ray images represented by orange (dotted line).

The aforesaid technique is quite reliable for visualization. However, it is expected that, by simple thresholding, voxel misidentification exists due to the noise and/or partial volume effect. The KNN classification algorithm was applied to increase the accuracy of the classification, as was described in Section 2.5.3. Figure 10a demonstrates how the two methods differ in segmentation. The histogram for the two phases in the wetted tablet is plotted based on the segmented label images using the KNN algorithm, as well as the histogram representing the whole tablet’s matrix with thresholding value (blue dashed line) separating the two phases. As it can be seen in Figure 10a, thresholding fails to classify the voxels with an intermediate gray-value. For instance, the tablet is completely dry at the first time step, while the thresholding method classifies 12% of voxels as wet.

Finally, the water penetration to the tablet was calculated at different time steps, as the ratio of the number of voxels classified as wetted region to the total number of voxels. Figure 10b compares the water penetration profile, calculated with the KNN method, with the released API measured by a UV spectrophotometer. Excellent agreement was found between the profiles for the first 2 h, but then the results diverged. The difference between the two graphs might be that at some point water is penetrated, but no API is dissolved yet.

## 4. Conclusions

In the current work, we introduced an X-ray tomography-based experiment to non-invasively monitor and characterize the dynamic behavior of pharmaceutical dosage forms during the dissolution process. A flow-through cell dissolution method was developed, capable of mimicking the in vitro dissolution process conducted by the paddle dissolution method, which can be easily positioned in an X-ray tomography setup. In order to evaluate the proposed technique, a model sustained-release 3D printed tablet comprising a mixture of insoluble polymer (polycaprolactone) and soluble drug particles (MPT) was utilized. The impact of flow rate on the dissolution rate of API from the tablet was studied, and 16 mL/min was chosen as a suitable flow rate. CsCl was used as a contrast agent to enhance the attenuation coefficient of the dissolution medium. Using such contrast agent is crucial, as in a system without the contrast agent, it was impossible to discriminate between the aqueous medium and the tablet’s matrix. The effect of CsCl on the released drug rate was investigated and found to be negligible. The API release rate obtained with the proposed technique was in a good agreement with that acquired by the standard in vitro dissolution test (with the similarity factor ($f_{2}$) equal to 77%). The method provided detailed information on the internal structure of the product during the dissolution process. In addition, it allowed visualization and quantification of water penetration into the sample.

In conclusion, the clear advantage of this work over the other existing approaches is the ability to investigate the dissolution mechanism without stopping the process and further sample preparation that is likely to affect the structure of the sample. Furthermore, the proposed method is demonstrated on sustained-release inert matrix tablets, yet it has the potential to be used to investigate other sustained-release formulations, e.g., hydrophilic matrix systems, which swell and partially erode during the dissolution process. However, these systems might be more challenging from a computational point of view, and great care must be taken when using a contrast agent or selecting a flow rate.

## Figures and Tables

**Table 1:** Setting for high quality and fast scans.

Scan Setting	High Quality Scans	Fast Scans
Voxels size (μm)	10.09	20.18
Number of projection (-)	1800	600
Number of averages (-)	3	2
Acquisition time (min)	8	2

**Table 2:** Overview of the similarity factor values for dissolution profiles obtained in different conditions.

In vitro Dissolution Experiment	Rate	$f_{2}$
Paddle dissolution method	100 rpm	reference
Flow-through cell method	16 mL/min	77%
Flow-through cell method	8 mL/min	35%
Flow-through cell method	4 mL/min	28%

## Data Availability

Not applicable.
